# Application of Mendelian randomization in thyroid diseases: a review

**DOI:** 10.3389/fendo.2024.1472009

**Published:** 2024-12-19

**Authors:** Zhonghui Li, Ruohan Wang, Lili Liu, Zonghang Jia, Peng Zhou, Qingqing He

**Affiliations:** ^1^ Shandong University of Traditional Chinese Medicine, First Clinical Medical College, Jinan, Shandong, China; ^2^ Department of Thyroid and Breast Surgery, The 960th Hospital of PLA Joint Logistics Support Force, Jinan, Shandong, China; ^3^ Department of Pathology, Dongying People’s Hospital (Dongying Hospital of Shandong Provincial Hospital Group), Dongying, Shandong, China

**Keywords:** Mendelian randomization, thyroid diseases, risk factors, etiological research, casual relationship

## Abstract

Thyroid diseases are increasingly prevalent, posing significant challenges to patients’ quality of life and placing substantial financial burdens on families and society. Despite these impacts, the underlying pathophysiology of many thyroid conditions remains poorly understood, complicating efforts in treatment, management, and prevention. Observational studies can identify associations between exposure variables and disease; however, they often struggle to account for confounding factors and reverse causation. Understanding disease occurrence, epidemiological trends, and clinical diagnosis, prevention, and treatment relies heavily on robust etiological research. Mendelian randomization, a method grounded in genetics and epidemiology, has been widely employed in studying the etiology of thyroid diseases, offering a solution to some of these challenges. This paper categorizes thyroid diseases into thyroid dysfunction and thyroid cancer, reviewing related Mendelian randomization studies. It further provides novel perspectives and approaches for investigating the mechanisms underlying thyroid diseases and designing intervention strategies.

## 1 Introduction

Thyroid disorders can be broadly categorized into those primarily managed with medical treatments and those requiring surgical intervention. Thyroid diseases treated medically include hyperthyroidism, hypothyroidism, and thyroid inflammation, which are predominantly characterized by abnormalities in thyroid function. In contrast, surgical treatments address conditions such as goiter and thyroid tumors. With the increasing prevalence of thyroid diseases in clinical practice, understanding their causes is vital for effective treatment. Randomized controlled trials (RCTs) are often considered the “gold standard” for establishing causality due to their high level of evidence; however, they are constrained by substantial implementation costs and ethical considerations (1). Observational studies, while useful for identifying associations between environmental factors and disease, frequently struggle to demonstrate causality. Mendelian randomization (MR) offers a method to evaluate causal relationships between exposures and clinical outcomes by utilizing genes strongly associated with specific traits as instrumental variables. These genes serve as proxies for exposure factors in regression models, allowing for an indirect analysis of causal relationships (2). MR has distinct advantages in avoiding confounding factors, reverse causation, and selection bias. This is because genes are randomly assigned during gamete formation, are minimally influenced by external environmental factors post-birth, and exhibit a unidirectional relationship with exposure (3). In the context of thyroid diseases, MR is primarily applied to etiological research, investigating the effects of environmental factors, individual traits, and microorganisms on the occurrence, progression, and resolution of specific thyroid conditions. These studies provide innovative insights for improving treatment strategies and prognosis. This paper systematically reviews the application of MR in the study of thyroid dysfunction and thyroid cancer, along with their associated risk factors.

## 2 Methods

Data for this study were obtained from PubMed through a comprehensive search using keywords such as “Mendelian randomization,” “thyroid,” “thyroid function,” and “thyroid cancer.” The most recent search was performed on 1 June 2024. Non-English studies, reviews, case reports, observational studies, RCTs, and other non-MR research were excluded. Literature meeting the criteria for Mendelian randomized trials with exposure or outcome elements related to thyroid diseases was included. Two researchers independently evaluated the titles and abstracts of all identified studies. Discrepancies during the screening process were resolved through discussion or consultation with a third party.

## 3 Results

**Figure 1:**
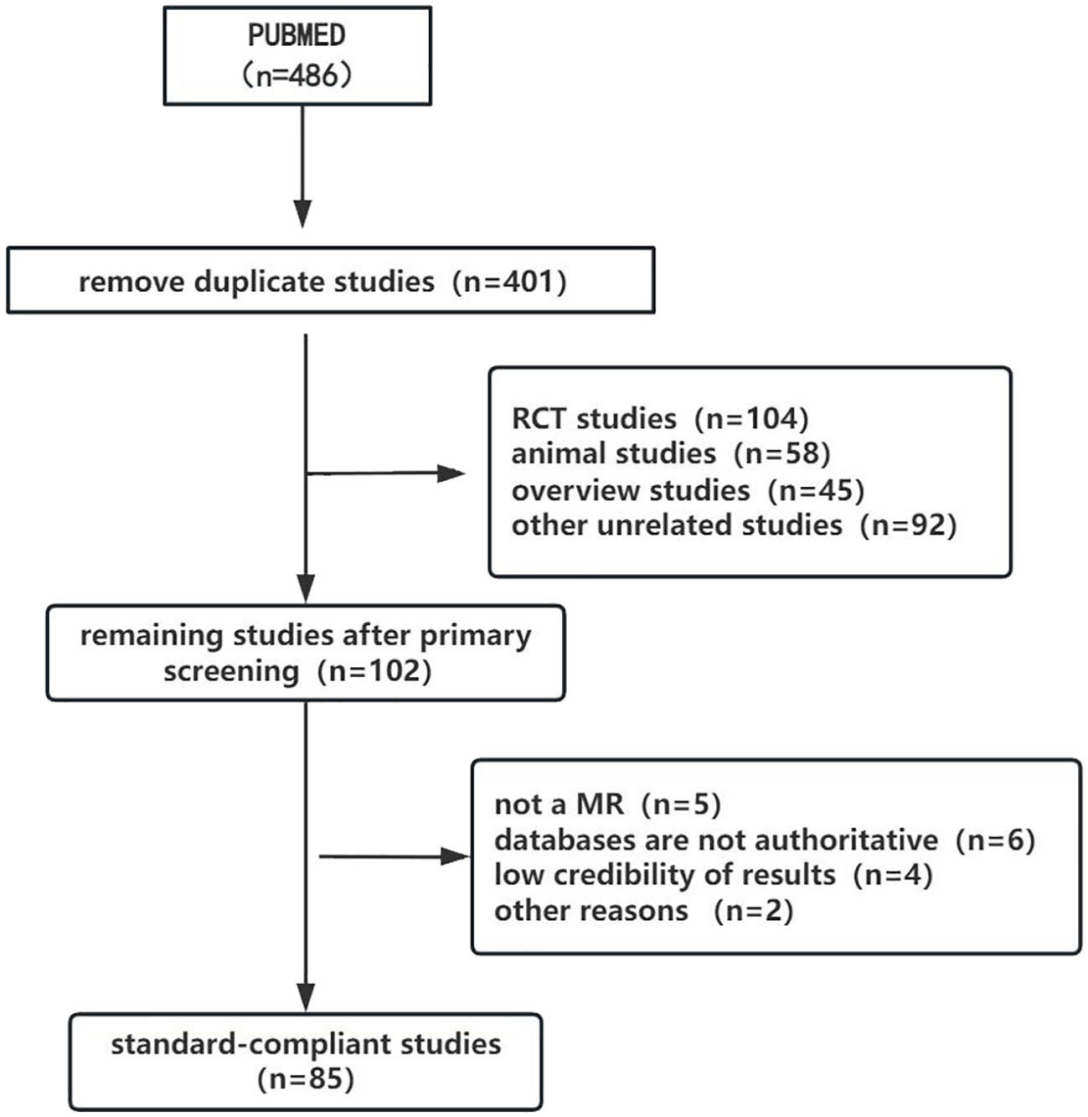
Literature screening flowchart.

The selection process for study inclusion and exclusion is illustrated in the flowchart (**Figure 1**). A total of 486 relevant articles were retrieved from the search, with 85 duplicates removed. After an initial screening of titles and abstracts, 299 articles were excluded, including RCTs, observational studies, reviews, and case reports that did not meet the inclusion criteria. Subsequently, 17 MR studies were excluded after full-text review due to incomplete data or other issues. Ultimately, 85 MR studies were included in the final review, comprising 67 studies on thyroid dysfunction and 18 on thyroid cancer.

### 3.1 Thyroid dysfunction

Thyroid dysfunction is one of the most common endocrine disorders encountered in clinical practice. It primarily involves abnormal thyroid hormone secretion, which can significantly impact both physical and mental health. Patients with thyroid dysfunction are typically categorized into two groups: those with hyperthyroidism and those with hypothyroidism. Disorders such as Graves’ disease, Hashimoto’s thyroiditis, and autoimmune thyroiditis are also major contributors to thyroid dysfunction. Previous studies employing MR have explored causal relationships between factors such as gut microbiota, immune responses, cytokines, thyroid disorders, and related conditions.

#### 3.1.1 Thyroid dysfunction and mental illness

Psychiatric disorders commonly encountered in clinical practice include various forms of depression (e.g., mild depression, major depression, and other subtypes), bipolar disorder, schizophrenia, anxiety disorders, and borderline personality disorder. Thyroid hormones are essential for neurocognitive development and overall neurological functioning. Consequently, the relationship between thyroid dysfunction and psychiatric conditions has been extensively studied over the past few decades (4, 5). Evidence suggests that mood disorders may disrupt the hypothalamic–pituitary–thyroid axis. For instance, individuals with major depression have been observed to exhibit a diminished thyroid-stimulating hormone (TSH) response to thyrotropin-releasing hormone (TRH) stimulation and a reduced nocturnal TSH surge (6).

Several MR studies have utilized free thyroxine (FT4) and TSH levels as exposure factors. These investigations found no association between genetically predicted FT4 and TSH levels and the risk of major depression, mild depression, schizophrenia, borderline personality disorder, or their subtypes. However, they observed that each standard deviation increase in FT4 levels reduced the overall risk of bipolar disorder by 11% and the risk of bipolar disorder type I by 13%. These results highlight the need for further clinical research on the role of thyroid hormones in bipolar disorder (7, 8). Another study demonstrated that higher TSH levels could decrease the risk of schizophrenia (9). Using hypothyroidism as an exposure factor, a study found significant genetic associations between hypothyroidism and anxiety disorders, schizophrenia, and major depression. Notably, major depression exhibited a causal effect, significantly increasing the risk of hypothyroidism (10). In a mediated Mendelian study, Zhang et al. (11) explored the relationships between major depression and thyroid diseases, identifying a significant risk of hypothyroidism, hyperthyroidism, and European-origin Hashimoto’s thyroiditis associated with major depression. Interestingly, alcohol consumption and antidepressant use were identified as mediators in reducing the risk of hypothyroidism. Furthermore, major depression was strongly linked to Hashimoto’s thyroiditis. These findings highlight the importance of larger RCTs to further explore the connection between thyroid function and psychiatric disorders.

#### 3.1.2 Thyroid dysfunction and cancer

Recent research has identified thyroid hormones as contributors to cell proliferation and differentiation, suggesting a pro-cancer effect in various malignancies (12, 13). Consequently, thyroid dysfunction has been categorized as a potential risk factor for both the development and prevention of cancer. Wang et al. (14) conducted MR studies to establish a causal relationship between hyperthyroidism and ovarian cancer, emphasizing the role of thyroid hormones in the prevention and management of female reproductive disorders. In contrast, other studies have demonstrated that hypothyroidism may have a protective effect against certain cancers, including malignant melanoma of the skin (15), gastric cancer (16), lung cancer (both squamous cell carcinoma and adenocarcinoma) (17), and liver cancer (18). The protective role of hypothyroidism in gastric cancer, in particular, has been linked to factors such as elevated cholesterol levels and other related mechanisms (19).

#### 3.1.3 Thyroid dysfunction and cardiovascular system diseases

The thyroid gland produces two key hormones, thyroxine (T4) and triiodothyronine (T3), which are essential for maintaining cardiovascular homeostasis by influencing heart rate, myocardial contractility, and vascular resistance. Thyroid function exerts a broad impact on the cardiometabolic system (20). Zhang et al. demonstrated that lower FT4 levels were significantly associated with increased carotid intima-media thickness, with apolipoproteins A-I and B acting as mediators in this relationship, highlighting the importance of monitoring thyroid function for early cardiovascular risk assessment (21). Additionally, reduced thyroid function has been linked to elevated cholesterol levels and an increased risk of cardiovascular diseases (22). Giontella et al. found that thyroid peroxidase antibodies (TPOAb) and hyperthyroidism were associated with a decreased risk of atrial fibrillation. MR analyses further revealed that TSH levels were inversely associated with systolic blood pressure and the risk of atrial fibrillation. Numerous studies have confirmed a causal relationship between hyperthyroidism and atrial fibrillation, with height identified as a significant mediator in these associations (23–25). The pituitary–thyroid–cardiac axis plays a key role in the development of cardiometabolic diseases. Wang et al. demonstrated causal associations between FT4 levels and conditions such as hypertension, hyperlipidemia, type 2 diabetes mellitus, ischemic heart disease, and non-rheumatic heart disease. Conversely, TSH levels were associated with heart failure and stroke (26). Using mediated MR analysis, Marouli et al. found that TSH levels were linked to a reduced risk of stroke, with atrial fibrillation serving as the primary mediator of this effect. Concurrently, Hashimoto’s thyroiditis was causally associated with an increased risk of coronary heart disease, with body mass index playing a significant role in this relationship (27). These findings emphasize the connection between thyroid function and cardiovascular diseases, suggesting that clinical focus on the cardiovascular health of patients with abnormal thyroid function is crucial for effective prevention of cardiovascular conditions.

#### 3.1.4 Thyroid dysfunction and gut microbiota

The thyroid–gut axis has been proposed as a potential link between thyroid diseases and the gut microbiota (28). A magnetic resonance imaging study revealed that individuals with hyperthyroidism had significantly higher levels of *Enterococcus* and significantly lower levels of *Bifidobacterium* and *Lactobacillus* in their intestines compared to healthy individuals (29). Xie et al. (30) conducted MR analyses on 211 microbial taxa and four thyroid characteristics, identifying 34 outcome-related gut microbiota. After adjustment, the class *Deltaproteobacteria* (id.3087) and phylum *Actinobacteria* (id.400) were found to be protective against hyperthyroidism and hypothyroidism, respectively. Liu et al. identified genera *Intestinimonas*, *Eubacterium brachy group*, *Ruminiclostridium 5*, and *Ruminococcaceae UCG004* as risk factors for decreased thyroid function, while phyla *Actinobacteria* and *Verrucomicrobia*, along with genera *Bifidobacterium* and *Lachnospiraceae UCG008*, were shown to have protective effects (31). In a study examining the causal relationship between gut microbiota, metabolites, and hypothyroidism, Liu et al. identified indolactic acid as the only metabolite associated with autoimmune hypothyroidism (32). Additionally, *Defluviitaleaceae* emerged as a promising probiotic for preventing influenza, subacute thyroiditis, and hypothyroidism. Zhang et al. demonstrated that this bacterium could causally inhibit the clinical pathway of influenza–subacute thyroiditis–hypothyroidism through its role in the gut microbiota (33). Liu et al. (34) applied MR techniques to investigate the connection between the gut microbiota and Graves’ disease, finding that *Veillonella*, *Bacteroides*, and the family *Bacteroidaceae* served as protective factors against the onset of Graves’ disease. Although these studies explore the associations between gut microbiota and thyroid function, further research is needed to clarify the precise effects of gut bacteria on thyroid health.

#### 3.1.5 Thyroid dysfunction and metabolism

A recent large-scale epidemiological survey conducted in China reported a strong association between thyroid disease and metabolic syndrome (35). According to an MR analysis investigating the relationship between metabolic syndrome and thyroid autoimmunity, the syndrome and its triglyceride component may act as potential protective factors against thyroid autoimmunity (36). Another MR study on thyroid function and metabolic syndrome found that higher FT4 levels were associated with a reduced likelihood of developing metabolic syndrome (37).

Diabetes mellitus, a group of metabolic disorders characterized by hyperglycemia, has also been linked to thyroid dysfunction. MR studies exploring the association between thyroid function and microvascular complications in diabetes indicate that elevated TSH levels within the reference range are associated with an increased risk of diabetic nephropathy and a reduced glomerular filtration rate. Similarly, lower FT4 levels and higher FT3/FT4 ratios within the reference range were linked to increased urinary albumin–creatinine ratios in diabetic patients (38). Zhao et al. used MR analysis to explore the causal link between thyroid function and type 1 diabetes mellitus, revealing a significant association between type 1 diabetes and hypothyroidism. Additionally, variations in thyroid function were shown to influence type 2 diabetes mellitus (39, 40).

Fatty liver disease, a common metabolic disorder, has also been associated with thyroid dysfunction. MR studies have established a causal relationship between thyroid function and fatty liver, showing a significantly higher incidence of fatty liver in individuals with hypothyroidism, while hyperthyroidism may serve as a risk factor (41). Circulating TSH levels have been linked to an increased risk of fatty liver disease, as demonstrated by Fan et al. (42).

The relationship between thyroid function and lipid metabolism has been investigated using MR analysis. Wang et al. found significant correlations between elevated TSH levels and serum total cholesterol and low-density lipoprotein levels. The FT3/FT4 ratio was also significantly associated with total cholesterol and low-density lipoprotein levels (43). Additionally, Zhang et al. observed that higher triglyceride–glycemic index levels were linked to hypothyroidism and subclinical hypothyroidism (44).

#### 3.1.6 Thyroid dysfunction and other immune disorders

Primary cholangitis, an autoimmune disease, is frequently accompanied by extrahepatic autoimmune conditions, with 73% of patients affected. MR analyses have identified bidirectional causality between primary cholangitis and seven extrahepatic autoimmune diseases, demonstrating that primary cholangitis increases the risk of autoimmune thyroid disorders (45). Another MR study linked primary cholangitis to thyroid dysfunction, particularly mild hypothyroidism (46). Additionally, a reverse MR analysis suggested that both hypothyroidism and hyperthyroidism may act as risk factors for primary cholangitis (47).

Systemic sclerosis, also known as scleroderma, is a connective tissue disease with complex and poorly understood autoimmune and genetic mechanisms. Han et al. applied MR analysis to show that systemic sclerosis was negatively influenced by hypothyroidism and autoimmune thyroiditis (48).

Systemic lupus erythematosus (SLE), an autoimmune connective tissue disease affecting multiple organs and systems, has also been linked to thyroid dysfunction. MR analysis conducted by Qin et al. identified a causal association between SLE and hypothyroidism, but not with hyperthyroidism. A reverse MR analysis further demonstrated a causal relationship between SLE and both hypothyroidism and hyperthyroidism. Another MR study also reported a higher incidence of hyperthyroidism and hypothyroidism in patients with SLE (49, 50).

Inflammatory bowel disease (IBD), an autoimmune condition characterized by immune system disturbances, includes ulcerative colitis and Crohn’s disease. Wu et al. used MR analysis to reveal that hypothyroidism significantly lowered the risk of IBD. TSH levels were associated with IBD, Crohn’s disease, and ulcerative colitis. Multivariate MR results further suggested that IP-10 may mediate the causal relationship between hypothyroidism and IBD (51).

Rheumatoid arthritis is an autoimmune disease characterized by erosive arthritis. Gu et al. used MR analysis to investigate the relationship between thyroid function and rheumatoid arthritis, revealing a familial susceptibility to hypothyroidism and an increased risk of rheumatoid arthritis. Reverse MR evidence further indicates that genetic susceptibility to rheumatoid arthritis is associated with an increased risk of both hypothyroidism and hyperthyroidism (52). Additional MR studies suggest a bidirectional causal relationship between rheumatoid arthritis and hyperthyroidism, hypothyroidism, Hashimoto’s thyroiditis, and autoimmune thyroiditis (53–55).

Myasthenia gravis, an autoimmune disorder resulting from impaired neuromuscular junction transmission, has also been linked to thyroid dysfunction. Wang et al. identified a causal relationship between Hashimoto’s thyroiditis, hypothyroidism, and myasthenia gravis through MR analysis, though FT4, TPOAb, and TSH levels were not associated with myasthenia gravis. Reverse MR analysis revealed a causal link between myasthenia gravis and Graves’ disease, while another study demonstrated causal relationships between myasthenia gravis and both hypothyroidism and hyperthyroidism (56, 57).

Autoimmune diseases arise from pathological autoimmune reactions that damage the body’s own tissues and cellular components, leading to tissue damage and organ dysfunction. The studies outlined above suggest that autoimmune thyroid diseases, as T-cell-mediated organ-specific autoimmune disorders, share a causal relationship with other autoimmune conditions. These findings highlight the importance of recognizing the connections between various autoimmune diseases to improve prevention, diagnosis, and management strategies.

#### 3.1.7 Thyroid dysfunction and blood disorders

In a two-sample MR study, Ellervik et al. explored the causal relationship between coagulation, fibrinolysis, and genetically predicted hyperthyroidism, hypothyroidism, TSH, and FT4. The study found that elevated TSH was associated with reduced vascular hemophilic factor and fibrinogen levels, while genetically predicted hyperthyroidism was linked to increased vascular hemophilic factor. Furthermore, hyperthyroidism and hypothyroidism were respectively associated with elevated and reduced tissue fibrinolysis plasminogen activator levels (58). An MR study examining the relationship between thyroid disorders and deep vein thrombosis indicated that hyperthyroidism slightly increased the risk of deep vein thrombosis, while no association was identified for other thyroid disorders (59). Another MR study supported a causal relationship between hyperthyroidism and an increased risk of venous thromboembolism (60). Regarding thyroid function and anemia, MR analysis demonstrated that both hypothyroidism and hyperthyroidism were linked to an increased risk of anemia. Genetic variants in the DIO3OS gene, associated with intracellular thyroid hormone regulation, were found to increase the risk of anemia (61). However, an MR study by Huang et al. found a significant causal relationship only between hypothyroidism and iron deficiency anemia, without evidence of a causal link between thyroid dysfunction and serum iron status markers (62). Additionally, autoimmune thyroid disease has been associated with pernicious anemia. However, this relationship is causally linked to impaired erythropoiesis rather than autoimmune hemolysis (63).

#### 3.1.8 Thyroid dysfunction and other diseases

##### 3.1.8.1 Head and face diseases

An MR study investigating the relationship between thyroid function and periodontitis found a causal link between genetic susceptibility to periodontitis and an increased risk of hypothyroidism. However, no evidence supported a relationship between periodontitis and the risk of hyperthyroidism or autoimmune thyroid disease (64). Chen et al. used an MR approach to evaluate the impact of thyroid function on sudden sensorineural deafness. The analysis showed that TSH levels were not a risk factor for sudden sensorineural deafness, but higher genetically predicted FT4 levels significantly reduced the risk (65). Chen et al.’s MR analysis indicated that hypothyroidism was causally linked to a higher risk of temporomandibular joint (TMJ) disorders, whereas no significant associations were found between FT4, TSH, or genetically predicted hyperthyroidism and TMJ disorders (66). MR analysis by Liu et al. demonstrated a positive correlation between genetically predicted hypothyroidism and an increased risk of cataract development. However, TSH levels, FT4, and hyperthyroidism were not significantly associated with cataracts (67). Li et al. examined the relationship between thyroid function and age-related macular degeneration, finding that genetic variations associated with higher FT4 levels within the normal range were linked to a higher risk of the disease (68).

##### 3.1.8.2 Lung diseases

Hong et al. identified a causal relationship between hypothyroidism and the development of sepsis, including asthma-related pneumonia and other types of sepsis, but no such association was found for hyperthyroidism (69). Zhang et al. employed MR methods to investigate the relationship between thyroid dysfunction and neocoronary pneumonia. Their findings indicated that thyroid dysfunction had no effect on susceptibility or severity of neocoronary pneumonia. A reverse MR study revealed that susceptibility to neocoronary pneumonia was a risk factor for hypothyroidism, but no evidence supported an association between any phenotype of neocoronary pneumonia and hyperthyroidism (70).

##### 3.1.8.3 Bone diseases

Qi et al., in an MR investigation, found a positive correlation between thyroid dysfunction and the risk of osteoporosis, with both hyperthyroidism and hypothyroidism contributing to an elevated risk of the condition (71). Additionally, a single-sample MR analysis investigating the causal relationship between serum TSH and fracture risk revealed that genetically increased serum TSH concentrations were associated with a reduced risk of fractures in men (72). Xiong et al. demonstrated a causal link between hypothyroidism and hallux valgus, with hypothyroidism increasing the incidence of this condition (73).

##### 3.1.8.4 Female or male diseases

In an MR investigation, Zhao et al. identified a link between hyperthyroidism and an increased risk of polycystic ovary syndrome (PCOS) (74). Another study using magnetic resonance imaging on thyroid function and PCOS found a significant correlation between TSH levels and the condition, suggesting that maintaining normal TSH levels may help prevent PCOS. Additionally, hyperthyroidism was also associated with the development of PCOS (75). Huang et al. demonstrated that hypothyroidism and TSH levels affect the genetically predicted risk of prostate enlargement and prostatitis (76). A summary of MR applications in thyroid dysfunction is presented in **Tables 1** and **2**.

**Table 1:** MR studies related to hyperthyroidism.

Risk factors	OR 95% CI	*p*	Relevance	PMID
Osteoporosis	1.077 (1.009,1.149)	<0.05	High risk	38834708
Vitiligo	1.12 (1.03,1.22)	0.01	Positive correlation	38807429
Major depression	1.57 (1.12,2.19)	0.009	Significant causal relationship	38788859
Ovarian cancer	1.000 (0.999,1.002)	0.857	Independence	38783224
Primary biliary cholangitis	1.19 (1.12,1.26)	1.13e-08	Positive correlation	38773317
Gastric cancer	0.976 (0.872,1.092)	0.669	Independence	38737547
Selenium	1.066 (0.976,1.164)	0.154	Independence	38708455
Heart failure	1.049 (1.013,1.087)	0.007	Positive correlation	38681769
Polycystic ovary syndrome	1.08 (1.0 2,1.13)	0.004	Positive correlation	38586452
Vitamin A	0.97 (0.95,1.00)	4.38e-02	Positive correlation	38919472
Statins	0.996 (0.993,0.998)	0.002	Significant correlation	38425755
Cataract	1.690e+02 (1.536e-01, 1.859e+05)	0.151	Independence	38375193
Temporomandibular disorders	1.10 (1.00,1.20)	0.042	Potential relevance	38368359
Serum 25 (OH) D	0.93 (0.695,1.256)	0.654	Independence	38259461
Atrial fibrillation	1.15 (1.11,1.19)	2.4×10^−14^	Correlation	30702347
	1.049 (1.016,1.083)	0.003	Correlation	38750912
Venous thromboembolism	1.124 (1.019,1.240)	0.019	Correlation	36277472
Systemic lupus erythematosus	1.045 (0.987,1.107)	0.11	Independence	36860870
Hallux valgus	0.496 (1.733e-05, 1.419e+4)	0.893	Independence	36967762
Prostatic hyperplasia	1.049 (0.990,1.111)	1.05×10^−1^	Correlation	37143736
Primary sclerosing cholangitis	1.001 (1.000,1.002)	0.000	Correlation	37928559
Gout	1.07 (1.01,1.12)	0.0314	Correlation	37932979
Rheumatoid arthritis	1.141 (1.050,1.239)	1.80e-03	Correlation	38090574
Deep vein thrombosis	1.0009	0.024	Correlation	38877527
Fatty liver	11.83 (2.9,22.54)	0.026	Correlation	38095388

**Table 2:** MR studies related to hypothyroidism.

Risk factors	OR 95% CI	*p*	Relevance	PMID
Toenail and blood selenium	0.85 (0.75,0.96)	0.009	Potential relevance	38840695
Osteoporosis	1.149 (1.083,1.219)	<0.0042	High risk	38834708
Uterine cancer	0.93 (0.87,1.01)	0.08	Independence	38818502
Major depression	1.40 (1.18,1.66)	0	Significant causal relationship	38788859
	1.43 (1.096, 1.866)	0.008	Significant correlation	38904036
Borderline personality disorder	1.09 (0.97,1.05)	0.30	Independence	38355654
Schizophrenia	1.01 (0.92,1.29)	0.61	Independence	38355654
	−0.06 (-0.10,-0.02)	0.004	Negative correlation	37589836
Ovarian cancer	1.000 (0.999,1.002)	0.857	Independence	38783224
Primary biliary cholangitis	1.10 (1.08,1.12)	7.02e-03	Potential relevance	38773317
Gastric cancer	0.936 (0.893,0.980)	0.006	Reduce risk	38737547
	0.93 (0.89,0.98)	0.003	Negative correlation	38904039
Polycystic ovary syndrome	1.06 (0.87,1.28)	0.559	Independence	38586452
Sepsis	1.097 (1.024,1.174)	0.008	Potential relevance	38586450
Vitamin C	0.69 (0.58,0.83)	1.05e-04	Protective factors	38919472
*Defluviitaleaceae*	0.043 (0.005,0.363)	0.018	Significant correlation	38476943
*Defluviitaleaceae_UCG_011*	0.38 (0.172,0.865)	0.021	Significant correlation	38476943
Systemic sclerosis	1.136 (0.977,1.321)	0.096	Independence	38431707
Myasthenia gravis	1.26 (1.08,1.47)	0.002	Correlation	38405140
Cataract	2.501 (1.325,4.720)	0.004	Potential relevance	38375193
Temporomandibular disorders	1.12 (1.05,1.20)	0.001	Significant correlation	38368359
Alopecia areata	1.40 (1.12,1.75)	3.03×10^−3^	Significant correlation	38292771
Indole lactic acid	1.592 (1.228,2.065)	0.036	Correlation	38239342
Renal function	1.05 (1.03,1.08)	3.3×10^−5^	Correlation	31910748
Anemia	1.12 (1.05,1.19)	1.31×10^−7^	Correlation	34514498
	1.101 (1.048,1.157)	0.001	Correlation	38857589
Primary cholangitis	1.068 (1.022,1.115)	0.003	Correlation	34956333
COVID-19	1.577 (1.065,2.333)	0.022	Correlation	36147565
Vascular endothelial growth factor	0.98 (0.84,1.15)	0.81	Independence	36159967
Liver cancer	0.997 (0.995,0.999)	0.016	Correlation	36246884
Venous thromboembolism	1.601 (0.693, 3.699)	0.27	Independence	36277472
Systemic lupus erythematosus	1.049 (1.020,1.079)	0.011	Correlation	36860870
Hallux valgus	2.838 (1.116,7.213)	0.028	Correlation	36967762
Prostatitis	0.853 (0.730,0.997)	4.6×10^−2^	Correlation	37143736
Lung adenocarcinoma	0.893 (0.813,0.981)	0.019	Correlation	37697335
Squamous carcinoma	0.888 (0.797,0.990)	0.032	Correlation	37697335
Gout	1.13 (1.03,1.21)	0.0336	Correlation	37932979
Rheumatoid arthritis	1.274 (1.210,1.342)	3.88e-20	Correlation	38090574
Malignant melanoma of the skin	0.987 (0.075,0.999)	0.041	Correlation	38093968
Inflammatory enteritis	0.761 (0.655,0.882)	0.001	Correlation	38872961
Periodontitis	1.24 (1.05,1.46)	0.012	Correlation	38193661
Fatty liver	7.62 (2.61,22.25)	<0.0001	Significant correlation	38095388
Type I diabetes	1.099 (1.018,1.186)	0.017	Correlation	35927830

### 3.2 Thyroid cancer

Cancer remains a significant threat to global health, with the mechanisms underlying most malignancies still poorly understood. This limited understanding contributes to delays in cancer diagnosis and treatment, exacerbating the global increase in cancer-related morbidity and mortality. Thyroid cancer, one of the most common endocrine tumors, has seen a global rise in incidence over the past three decades due to the expanded use of diagnostic imaging techniques and ultrasound-guided fine-needle aspiration (77, 78). Despite the increasing incidence, the mortality rate of thyroid cancer has remained relatively stable. Risk factors for thyroid cancer include environmental pollution, unhealthy lifestyle choices, and metabolic syndrome; however, these factors do not fully explain the disease’s pathogenesis. Thus, identifying additional modifiable risk factors is crucial for improving prevention and management strategies.

#### 3.2.1 Thyroid cancer and other cancers

Thyroid and breast cancers are the two most common types of cancer among women worldwide, both influenced by hormonal changes and driven by similar endocrine signals. Emerging evidence suggests a potential bidirectional pathogenic relationship between these two cancers (79). Several studies have explored the relationship between thyroid cancer and various subtypes of breast cancer. Some have identified a causal link between ER-positive breast cancer and an increased risk of thyroid cancer (80, 81). Additional MR studies have examined the relationship bidirectionally, revealing potential connections (82). One study suggested an association between thyroid cancer and ER-negative breast cancer as well (83). Furthermore, an MR study evaluating the causal relationship between prostate cancer and 12 other types of cancer found that thyroid cancer might increase the risk of prostate cancer (84).

#### 3.2.2 Thyroid cancer and gut microbiota

An MR study analyzing the causal relationship between gut microbiota and thyroid cancer identified nine microbial taxa associated with thyroid cancer risk. Among these, *Christensenellaceae*, family *Victivallaceae*, genus *Methanobrevibacter*, genus *Ruminococcus 2*, genus *Subdolinglanlum*, and phylum *Verrucomicrobia* were linked to an increased risk of thyroid cancer, while class *Betaproteobacteria*, family *XI*, and genus *Sutterella* were associated with a reduced risk (85). Zhu et al. identified seven microbial taxa significantly associated with thyroid cancer through MR analysis: genera *Butyrivibrio*, *Fusicatenibacter*, *Oscillospira*, *Ruminococcus 2*, and *Terrisporobacter* were identified as risk factors, whereas genera *Olsenella* and *Ruminococcus UCG004* were associated with a reduced risk. Reverse MR analysis indicated that thyroid cancer may cause an increase in genus *Holdemanella* and a decrease in *Bacillales* (86). Quan et al. conducted an MR study suggesting that genus *Ruminiclostridium 9*, class *Mollicutes*, genus *Ruminococcaceae UCG004*, genus *Paraprevotella*, and phylum *Tenericutes* were associated with an increased risk of differentiated thyroid cancer, while phylum *Actinobacteria* was linked to a reduced risk (87). Similarly, Hu et al. identified four microbial communities, class *Mollicutes*, phylum *Tenericutes*, genus *Eggerthella*, and order *Rhodospirillales*, as being associated with the risk of differentiated thyroid cancer (88).

#### 3.2.3 Thyroid cancer and other diseases

##### 3.2.3.1 Mental illness

Numerous MR studies have demonstrated a positive causal relationship between psychiatric disorders, particularly schizophrenia and depression, and thyroid cancer (89–91). Yuan et al. investigated the genetic association between 13 types of cancer and schizophrenia. Their findings revealed that individuals with schizophrenia have a higher likelihood of developing thyroid cancer (92).

##### 3.2.3.2 Rosacea

Chronic inflammation is known to accelerate cancer development, and rosacea, a persistent inflammatory skin condition, has been associated with an increased risk of various malignancies. Li et al. conducted a comprehensive study on the relationship between rosacea and cancer in American women, concluding that individuals with a history of rosacea are more likely to develop thyroid cancer (93). However, a bidirectional, two-sample MR study by Luo et al. found no causal relationship between rosacea and thyroid cancer (94).

##### 3.2.3.3 COVID-19

The MR investigation by Xu et al. found no evidence supporting the hypothesis that severe COVID-19 cases increase the risk of thyroid cancer or that susceptibility to COVID-19 elevates thyroid cancer risk (95).

##### 3.2.3.4 Cardiovascular diseases

Gao et al. used MR analysis to explore the relationship between thyroid cancer and various cardiovascular diseases. Their results indicated a potential causal relationship between thyroid cancer and heart failure. However, no association was identified between thyroid cancer and other cardiovascular conditions, including atrial fibrillation, coronary artery disease, myocardial infarction, and ischemic stroke (96). A summary of the application of MR in thyroid cancer is presented in **Table 3**.

**Table 3:** MR studies related to thyroid cancer.

Risk factors	OR 95% CI	*p*	Relevance	PMID
Rosacea	1.04 (0.90,1.20)	0.59	Independence	38769705
Family *Christensenellaceae*	1.664 (1.103,2.511)	0.015	Correlation	38495789
Genus *Methanobrevibacter*	1.505 (1.049,2.159)	0.027	Correlation	38495789
Genus *Ruminococcus 2*	1.846 (1.261,2.704)	0.002	Correlation	38495789
Class *Betaproteobacteria*	0.522 (0.310,0.879)	0.015	Correlation	38495789
Genus *Sutterella*	0.596 (0.381,0.933)	0.024	Correlation	38495789
Heart failure	1.00134 (1.00023,1.00244)	7.02e-03	Potential causality	38773317
Major depression	3.956 (1.177,13.299)	0.026	Potential relevance	38594691
Schizophrenia	1.532 (1.123,2.088)	0.007	Potential relevance	38594691
	0.950 (0.849,1.061)	0.3617	Independence	38434685
	1.5482 (1.1112,2.1569)	0.0098	Potential relevance	38044984
	1.575 (1.048,2.365)	0.0285	Correlation	36138824
	1.543 (1.023,2.328)	0.039	Correlation	38843742
Bipolar disorder	0.919 (0.460,1.836)	0.811	Independence	38594691
Depression	1.14 (0.53,1.43)	0.458	Independence	35361153
	3.573 (1.068,11.953)	0.039	Correlation	38843742
CD62L	0.953 (0.930,0.976)	1.005×10^−4^	Correlation	38953025
Copper	0.88 (0.65,1.20)	0.414	Independence	38496794
Zinc	0.87 (0.64,1.19)	0.399	Independence	38496794
Calcium	1.12 (0.84,1.51)	0.431	Independence	38496794
Vitamin C	0.95 (0.87,1.03)	0.219	Independence	38496794
Vitamin D	0.89 (0.61,1.30)	0.561	Independence	38496794
Iridium	1.18 (0.81,1.73)	0.485	Independence	38496794
PCSK9 inhibitor	1.12 (0.65,1.59)	0.64	Independence	38275613
Glutamine	0.577 (0.421,0.790)	6.14×10^−4^	Negative correlation	38187059
COVID-19	1.061 (0.575,1.956)	0.850	Independence	38162775
Hyperthyroidism	0.95 (0.46,1.96)	0.897	Independence	32215913
Hypothyroidism	0.70 (0.51,0.98)	0.038	Correlation	32215913
Primary cholangitis	1.106 (1.019,1.120)	0.042	Correlation	34956333
Systemic lupus erythematosus	2.31 (1.55,3.45)	4.31×10^−5^	Correlation	35600353
Dried fruit intake	0.1854 (0.0002,195.8003)	0.6352	Independence	35923199
ER-positive breast cancer	1.135 (1.006,1.279)	0.038	Correlation	37288296
ER-positive breast cancer	1.6752 (1.1110,2.5258)	0.014	Correlation	38082224
ER-positive breast cancer	1.019 (1.001,1.038)	0.043	Correlation	3795501
Triple-negative breast cancer	1.135 (1.006,1.279)	0.177	Independence	37288296
Iga nephropathy	1.191 (1.131,1.253)	0.001	Significant correlation	37670254
High-density lipoprotein	0.75 (0.60,0.93)	0.01	Correlation	37780617
Gout	1.03 (0.98,1.09)	0.297	Independence	37932979
Chemokine CCL20	0.76 (0.61,0.95)	0.015	Correlation	38075694
Congenital iodine deficiency	1.374 (1.110,1.700)	<0.05	Correlation	38129522
Prostatic cancer	1.12 (1.08,1.16)	0.008	Correlation	37740849

## 4 Summary and limitations

Etiological research plays a crucial role in identifying risk factors essential for treating, preventing, and improving patient outcomes. MR analysis offers a valuable tool by bypassing the labor-intensive data collection process typical of observational studies, instead relying on genetic information from study subjects. By effectively establishing causal relationships between exposure factors and outcomes, while avoiding issues such as reverse causality and confounding variables, MR analysis has gained widespread application in thyroid research and broader medical fields. Given the diverse manifestations of thyroid disorders, MR analysis is frequently employed to identify less obvious risk factors associated with these conditions.

Despite its significant contributions, MR analysis has limitations that warrant further refinement. One key issue is linkage disequilibrium (LD), which arises when alleles segregate in a manner deviating from Mendel’s second law. This interference between related genetic variants can introduce bias into results, as the strength of association between instrumental variables and outcomes is critical. To address this, MR can be applied in populations with different LD structures. Another challenge is gene pleiotropy, where a single gene influences multiple phenotypic traits. Type I pleiotropy, in which a single locus directly affects multiple phenotypes, can complicate study outcomes due to high correlations between traits (97, 98). This issue can be identified and addressed through sensitivity analyses. Finally, the genetic data used in MR analysis often come from biobanks, such as the UK Biobank. This reliance introduces potential biases due to allele frequency and the prevalence of associated disorders within the study population. Variability in instrumental variables, exposures, and outcomes across diverse groups can result in substantial errors. These issues can be minimized by restricting the study population’s background or, when unavoidable, using adaptive switching methods to reduce errors.

Although significant progress has been made in understanding the risk factors for thyroid disease, effective therapies and prevention strategies have yet to be fully implemented. Current thyroid research still faces gaps in understanding the pathophysiology of complex disorders and the translation of findings into clinical practice, such as identifying therapeutic targets and advancing gene therapy. To address these challenges, an innovative approach for applying MR in thyroid research could involve integrating GWAS with multi-omics data, including proteomics and metabolomics, to identify drug-targeting proteins. Furthermore, genes could serve as instrumental variables to pinpoint pathogenic genes and potential targets for intervention.

This review has certain limitations. First, it focused on studies published within a specific time frame and database, potentially excluding relevant articles. Second, it was not a systematic evaluation, and the eligibility criteria were not explicitly defined. Despite these limitations, this review categorizes and discusses findings from MR studies related to thyroid disorders, offering valuable insights that could inform prevention and treatment strategies in clinical practice.
